# Managing Early Childhood Caries with Atraumatic Restorative Treatment and Topical Silver and Fluoride Agents

**DOI:** 10.3390/ijerph14101204

**Published:** 2017-10-10

**Authors:** Duangporn Duangthip, Kitty Jieyi Chen, Sherry Shiqian Gao, Edward Chin Man Lo, Chun Hung Chu

**Affiliations:** Faculty of Dentistry, The University of Hong Kong, Hong Kong, China; dduang@hku.hk (D.D.); kjychen@hku.hk (K.J.C.); gao1204@hku.hk (S.S.G.); edward-lo@hku.hk (E.C.M.L.)

**Keywords:** child, dental caries, dentine, primary teeth, fluoride(s), therapeutics, silver compounds, minimally invasive dentistry

## Abstract

Early childhood caries (ECC) is a significant global health problem affecting millions of preschool children worldwide. In general, preschool children from families with 20% of the lowest family incomes suffered about 80% of the ECC. Most, if not all, surveys indicated that the great majority of ECC was left untreated. Untreated caries progresses into the dental pulp, causing pain and infection. It can spread systemically, affecting a child’s growth, development and general health. Fundamental caries management is based on the conventional restorative approach. Because preschool children are too young to cope with lengthy dental treatment, they often receive dental treatment under general anaesthesia from a specialist dentist. However, treatment under general anaesthesia poses a life-threatening risk to young children. Moreover, there are few dentists in rural areas, where ECC is prevalent. Hence, conventional dental care is unaffordable, inaccessible or unavailable in many communities. However, studies showed that the atraumatic restorative treatment had a very good success rate in treating dentine caries in young children. Silver diamine fluoride is considered safe and effective in arresting dentine caries in primary teeth. The aim of this paper is to review and discuss updated evidence of these alternative approaches in order to manage cavitated ECC.

## 1. Background

Early childhood caries (ECC) is the term used to describe the presence of decayed, missing or restored teeth in the primary dentition of children younger than six years old [1]. It is considered one of the most prevalent diseases in childhood, affecting 60% to 90% of children globally [2]. Although fluoridated toothpaste and the continued use of fluoride in various forms are effective in caries prevention [3], ECC is still prevalent among children in disadvantaged communities in both developing and developed countries [4]. Untreated decayed teeth causes difficulties with sleeping and eating and affects children’s growth and development [5]. The International Federation of Dentistry (FDI) reported that ECC is one of the main reasons for school absence in several countries [5]. It can progress rapidly, resulting in pain and infection and affecting child’s oral health related quality of life [6]. Such problems could become serious and even life threatening [7]. Despite the decline of dental caries in adults, an increase of caries prevalence among preschool children has occurred in many countries [8]. In Southeast Asia, dental caries is highly prevalent in preschool children. Their median caries prevalence was 79%, and their caries experience in terms of dmft (decayed, missing and filled primary teeth) score was 5.1 [9]. The situation seems more severe in low-income countries. For instance, caries prevalence among 6-year-old children in Cambodia was as high as 91%, and their mean dmft score was 7.9 [10]. In Vietnam, high caries prevalence (74%) and a large proportion of untreated decayed teeth (95%) were observed [11]. China and India are the two most populated countries in the world. Their caries prevalence of preschool children is relatively high at 66% in China [12] and 63% in India [13] compared with that in high-income countries, such as the USA (23%) [14] and the UK (28%) [15].

Early and regular dental visits can slow down the onset of dental caries in very young children and may result in fewer subsequent treatment visits and reduce the cost of treatment [16]. Despite the benefit of early prevention, access to dental care remains very low in deprived communities. These problems may be due to the poorly developed oral healthcare systems. Many countries in Asia and Africa have severe shortages of dental health personnel [17]. Thus, the capacity of the healthcare system is restricted to emergency treatment and pain relief. In Africa, the dentist-to-population ratio is approximately 1:150,000 in contrast to about 1:2000 in some developed countries [17]. It seems that the prevailing methods for caries prevention and treatment that the experts in high-income countries suggest are neither affordable nor available in low-income countries.

Aetiology of tooth decay is well researched, and thus it is theoretically preventable. Caries management for preschool children may differ from that for adults, as an atraumatic approach for children can slow the progression of caries so that the arrested decayed tooth exfoliates before causing oral pain. However, a very low proportion of paediatric dentists to child populations exists, especially in developing countries. It is impossible to handle such situations with the limited number of dentists in this specialty alone. The approach for tackling the heavy burdens of tooth decay must be effective, low cost and technically insensitive. In addition, the approach must be simple to use, as more general dentists may therefore adopt it, and it may be implemented in kindergartens for children in need, thus increasing access to dental care. The intent of the present review is to help dentists to make decisions regarding which evidence-based approaches are effective and feasible in managing cavitated ECC. This paper examines and summarises the available scientific literature in this regard. A PubMed search of the studies published in English using the medical subject heading (MeSH) terms of “dental atraumatic restorative treatment” or “fluorides” or “silver compounds” was performed.

## 2. Restorative Approach to Managing Cavitated ECC

The extensive restoration of primary teeth is the conventional dental treatment for young children with ECC. It has received support from dental association policies, insurance coverage, and accreditation training requirements in dentistry. However, given the comprehension of the determinants of salutary health behaviours, the progression of dental caries persists even after tooth repair. New dental materials and techniques for restoring decayed teeth have been developed. However, despite much improvement in dental materials and healthcare technology, children from families with low socioeconomic status may not benefit much. The longevity of dental restorative materials in the primary dentition was investigated, and studies showed unsatisfactory results [18,19]. The failure rates of amalgam and composite fillings could be up to 58% and 62%, respectively, whereas conventional glass ionomer cements showed a 33% success rate over five years [18]. A recent systematic review of the dental materials and techniques for restoring primary teeth in young children revealed that limited evidence exists for making decisions regarding the appropriate choice for dental practitioners [20]. The success of two-surface restorations varied considerably, ranging from 18% to 80% [20]. Stainless steel crowns are generally recommended for restoring fragile primary teeth after pulp therapy or multiple carious surface lesions [21]. Nevertheless, few dentists have adopted this technique as a routine due to the perceived clinical difficulty and the high cost of materials [22]. Operative treatment in preschool children was found to be unpopular among general dental practitioners. Less than 15% of tooth decay was restored in 5-year-old children in the UK [23]. For apprehensive young children with severe ECC, restorative procedures usually require well-trained dental health personnel and sophisticated clinical procedures.

The Hall technique can be a minimally invasive operative technique for managing cavitated lesions in young children [24]. It has been proposed as a modified technique for stainless steel crowns that involves sealing the carious lesions of primary molars with stainless steel crowns without caries removal and crown preparation. Recently, a Cochrane review concluded that a patient’s discomfort following the Hall technique was lower than that associated with conventional restorations (risk ratio 0.56, 95% confidence interval (CI): 0.36–0.87) [25]. Although the Hall technique is more simplified than the conventional restorative technique, the cost of materials and the operation time required may be obstacles to its adoption in a community-based program.

### Atraumatic Restorative Treatment (ART)

Restorative care in the primary dentition is essential for maintaining adequate space and functional conditions until tooth exfoliation. Conventional dental restorative treatment with composite resin and amalgam required sophisticated procedures and equipment in a clinic setting. Atraumatic restorative treatment (ART) was initially developed to provide effective restorative treatment in developing countries where electricity may not be available [26]. The use of ART has increased gradually and remains high, particularly in disadvantaged communities [27]. It is a pain-free restorative procedure that involves no local anaesthesia or drilling. The ART approach involves caries removal through the use of hand instruments, followed by restoration with highly viscous glass ionomer cement (GIC), which provides chemical adhesion to the tooth surface, fluoride release and biocompatibility. The adjacent pits and fissures can also be sealed simultaneously using glass ionomer cement inserted under finger pressure [28]. During the placement of a restoration, ART is easy to handle because a single increment is needed. It is suggested that ART provides an alternative approach to the management of ECC in a primary dental care setting [29]. Based on the summary of several systematic reviews of ART (Table 1), evidence exists that minimally invasive approaches with ART are beneficial in managing primary decayed teeth [30,31].

Numerous studies have been published on the longevity of ART in both primary and permanent teeth. A systematic review and meta-analysis on the quality of ART showed high successful rates of ART on single-surface cavities in primary teeth (86% survival rates over three years) [32]. However, the survival of ART restorations in multiple-surface cavities in primary teeth was unsatisfactory; a higher annual failure rate (17%) was reported compared with those for single-surface cavities (5%) [32]. Similarly, the survival rates of ART restorations with highly viscous GIC in primary teeth were 93% and 62% over two years for single- and multiple-surface ART, respectively [30]. No statistically significant difference in the survival rates of class I (pit and fissure, on occlusal, buccal, and lingual surfaces of molars) ART and amalgam restorations in in primary teeth was found (relative risk (RR) 1.07; 95% CI: 0.91–1.27; *p*  =  0.39) after 24 months [33]. ART with highly viscous GIC presented similar success rates for conventional restorations using amalgam or composite for occluso-proximal cavities [34]. Recently, a systematic review of restorative approaches in preschool children supported the use of minimally invasive approaches, such as ART, in preschool children [20].

Besides the clinical outcomes that clinicians determined, patient-based outcomes of ART studies were also reported. The ART procedure produced less anxiety when compared with other restorative procedures using a dental drill [35]. Prior to ART treatment, the initial anxiety of preschool children was greater, and since the treatment has been adopted, the level of anxiety has decreased [36]. A high acceptability rate for the ART procedure was found in young children [37,38]. A cohort study by Lo and colleagues concluded that 93% of the study children reported no dental pain during the ART procedure, and 86% of them were willing to receive the treatment again [37]. In addition, ART restoration was found to be a cost-effective restorative option compared with amalgam and composite restoration although ART was performed in a clinic setting [39]. Similarly, a study about the cost-effectiveness of the ART-based approach compared with standard care (by referral to paediatric dental specialist care) was conducted in Australia [40]. It was concluded the ART-based approach to managing ECC is worthwhile, since it allows more treatments with fewer referred cases. The probability that ART can lead to cost savings is 63% [40].

The concept of ART is now receiving support from a recent report from the International Caries Consensus Collaboration meeting, indicating that less invasive treatment should be adopted in addition to preserving the dental tissue and delaying the cycle of restoration [41]. Following this recommendation, carious tissue should be removed merely to promote conditions for durable dental restorations. Due to its effectiveness and feasibility, the use of ART can be a vital component in dental public health services for young children and those with special needs [28]. In spite of its clinical success, the use of ART still requires some clinical skills and relatively expensive dental materials [27]. A summary of the systematic reviews of ART in managing primary decayed teeth is presented in Table 1. Although several systematic reviews reported similarities regarding the high success rates of ART on single-surface lesions, it should be noted that most of the included studies investigated the survival rates of ART in primary teeth in school children. For the current review, a list of randomized clinical trials of ART conducted in children aged six years or younger is shown in Table 2. More well-designed randomized clinical trials are needed to confirm the effectiveness of ART in managing ECC.

## 3. Non-Restorative Approaches to Managing Cavitated ECC

The effectiveness of various fluorides in controlling dental caries has been categorized via different methods of delivery, such as community based, professionally administered and self-applied [55]. Community-based water fluoridation is proven to be a beneficial measure for caries reduction in several countries [56]. The advantage is that no cooperation effort of an individual is required. For deprived child populations, community-based fluoridation measures remain the most equitable and effective strategy for caries control in children.

Regarding the self-applied fluoride, fluoridated toothpaste is the most commonly used form of fluoride delivery. Several reviews showed its anti-caries effects [57,58]. Significant factors influencing its efficacy include the formulation of dentifrice as well as brushing behaviours, such as brushing frequency, brushing time and post-brushing rinsing practices [58]. Despite fluoride’s beneficial effect in preventing caries, studies on the effectiveness of fluoride toothpaste on arresting dentine caries are limited in the literature. An in situ study confirmed that removing plaque daily with fluoride toothpaste (1100 ppm) in combination with 2% NaF varnish affected the distribution of mineral and probably arrested the progression of caries [59]. Arrested caries in young children was presented as an additional finding in a study that was initially planned to examine the preventive effect of a tooth brushing exercise using toothpaste (1000 ppm F) [60]. The three-year results showed that around half of the proximal lesions of the anterior teeth had become arrested. This provides some evidence that the use of simple oral health programs can stabilize or control caries in communities where access to restorative treatment is limited. Another study carried out on 300 Brazilian school children with enamel lesions found that supervised tooth brushing in combination with or without APF gel could arrest white spot lesions [61]. Poor oral hygiene increased the chance of keeping enamel lesions active. Daily tooth brushing using fluoridated toothpaste in combination with professionally applied topical fluorides can halt the progression of non-cavitated carious lesions [62]. Oral health education with professional preventive care or with supervised tooth brushing can also reduce caries prevalence in children. Other interventions, such as improving children’s diets or providing oral health education alone, had only a limited impact [63].

Professionally applied topical fluorides are effective in preventing and arresting ECC [64]. A Cochrane systematic review concluded that fluoride varnish has a substantial caries-inhibiting effect in primary teeth with the pooled decayed, (extracted/missing) and filled primary surfaces (d(e/m)fs) prevented fraction estimate of 37% (95% CI: 24–51%; *p* < 0.0001) [65]. Similarly, another systematic review supported the use of fluoride varnish in caries prevention in preschool children due to its efficacy and safety [66]. For a caries-arresting effect, most of the studies focused on inhibiting initial enamel caries [67,68]. It is suggested that the application of 5% sodium fluoride varnish can remineralise early enamel caries in primary teeth [45,68]. However, few studies on the caries-arrest effectiveness of fluoride varnish in dentine cavitated caries lesions were published [52,54], and these results were unfavourable compared with those of the silver diamine fluoride (SDF) application.

### 3.1. Silver Diamine Fluoride (SDF)

Among the various professionally applied topical fluorides, the use of silver diamine fluoride is currently gaining more popularity due to the favourable results of arresting dentine caries in primary teeth [52,53,54]. Actually, silver compounds have been developed and used for various purposes in dentistry since the 1940s for caries prevention [69], cavity sterilization [70] and dentine desensitizer [71]. SDF was accepted as a therapeutic agent in Japan more than 40 years ago [72,73]. In Australia, minimal intervention by using 40% neutral silver fluoride solution has been adopted for caries control in primary teeth since the 1970s [74]. The use of silver fluoride solution followed by stannous fluoride was found to be beneficial for stopping caries progression in the primary molars of children [75].

In China and Hong Kong, SDF has been successfully adopted for arresting dental caries for many years [20,52]. A clinical trial in preschool children confirmed that 38% SDF is more effective in arresting dental caries in primary teeth compared with 5% NaF varnish. No additional benefit of caries arrest was found when removing soft caries prior to the application of either SDF or NaF. Another study comparing the effectiveness of GIC restoration and SDF concluded that no differences existed in the caries-arrest effectiveness of 38% SDF applied once a year and the restoration with glass ionomer cement in primary teeth after two years [50]. More recently, a clinical trial conducted in Hong Kong found that three-weekly applications of SDF is as effective as an annual application of SDF in arresting caries for mobile populations after 18 months [54]. Different concentrations of SDF and different periodicities of SDF application were investigated. It was shown that 38% SDF showed a more beneficial effect than 12% SDF did, and when applied semi-annually rather than annually [53]. 

In Latin America, a few clinical trials of SDF were found in the literature. The effect of SDF on initial lesions in erupting permanent molars was equally efficient in controlling occlusal caries after 30 months compared with the cross tooth-brushing technique and GIC sealants [76]. Another study revealed that 30% SDF was more effective (caries arrest rate 67%) than the interim restorations with glass ionomer cement (caries arrest rate 39%) in arresting dentine caries in primary teeth in preschool children [51]. The nano silver fluoride (NSF) was recently developed and investigated in 130 primary teeth with active caries in school children in Brazil. Similar findings were reported: 67% caries arrest in the NSF group and 35% in the control group after one year [77]. The authors concluded that NSF was effective and claimed that it did not stain the carious lesions after application. However, further studies are required to refute or confirm NSF treatment. 

In Nepal, a single application of 38% SDF was effective in both the anterior and posterior primary teeth of children ages 3–9 years, but the treatment effectiveness in caries arrest decreased over time [78]. In the USA, the alternative protocol involving the use of 25% silver nitrate followed by 5% NaF Varnish on carious lesions was proposed and found to be well accepted by patients. The results of caries arrest treatment were also satisfactory [79]. Because SDF is unavailable in many countries, the adjunctive application of 5% NaF and 25% silver nitrate may arrest ECC [80]. Further study is required to warrant or refute these findings.

The summary of systematic reviews of the effectiveness of SDF is presented in Table 1. Following the 38% SDF treatment in primary teeth, a high caries arrest rate percentage of 81% (95% CI: 68–89%) was reported in a systematic review [46]. SDF is currently considered an effective preventive and therapeutic agent for caries management in preschool children due to its safe, simple, low-cost and effective treatment [44,45]. The use of SDF for treating cavitated carious lesions is in accordance with the clinical recommendation of the International Caries Consensus Collaboration meeting indicating that dental professionals should control caries via plaque removal, preserve dental hard tissues and retain natural teeth by avoiding the restorative cycle as much as possible [41]. Details of randomized clinical trials of SDF conducted in preschool children is presented in Table 2.

Even though the mechanism of SDF is not clearly understood, several laboratory studies reported the possible mechanisms, such as inhibiting the process of demineralisation and conserving collagen from degradation [81], antibacterial effect on oral biofilms [82] and the increased microhardness of dentine lesions after SDF application [83]. A recent systematic review concluded that SDF is a bactericidal dental agent that inhibits the growth of cariogenic bacteria [84]. It can inhibit demineralisation, promote the remineralisation of enamel and dentine as well as hamper the degradation of the dentine collagen. If the application of SDF is widely adopted among general dental practitioners, the workforce in dealing with the heavy burden of ECC will be expanded. Consequently, it will reduce the harmful impact of oral health problems in preschool children from low income families who usually have limited access to conventional dental care. Up to now, no major side effects have been found in clinical studies of SDF except for black or brown staining on treated lesions [52,53,54]. For parents or children with aesthetic concerns, the black stain was found to be the most-cited barrier to adopting the SDF treatment [85]. Thus, staining on posterior teeth is found to be more acceptable than staining on anterior teeth is [86]. Even though staining on anterior teeth was a concern, most parents in the US preferred SDF treatment to advanced pharmacological approaches, such as general anaesthesia [86]. Prior to the implementation of oral health promotion with SDF, the benefits, side effects and treatment options should be discussed. Dental practitioners need to acknowledge parental acceptability and preference to plan adequately for treatment adoption in managing ECC.

Oftentimes, social determinants, such as poverty, can influence the success of an oral health program [87]. Fortunately, the results of SDF studies showed no negative effect of social factors on arresting cavitated dentine lesions [53,54]. This suggests that use of SDF would remain highly effective in community oral health intervention programs in disadvantaged communities where untreated ECC is usually prevalent. It should be noted that oral hygiene and the presence of plaque on a lesion are paramount for the success of various caries arrest treatments [50,54]. To enhance its treatment efficacy in managing ECC, training on effective dental plaque control must be provided in the SDF program. It is also crucial to empower all stakeholders to improve the children’s oral hygiene, such as by encouraging parents to supervise and assist their children in tooth brushing. Kindergarten teachers should also reinforce oral hygiene practice in schools.

### 3.2. Other Non-Fluoride Agents

Although many studies investigated the caries preventive effect of non-fluoride agents, such as chlorhexidine [88], casein phosphopeptide-amorphous calcium phosphate (CPP-ACP) [89], and xylitol [90], insufficient evidence exists to support the use of these agents as a therapeutic agent to arrest cavitated dentine caries in preschool children [44].

Xylitol is a non-fermentable sugar alcohol which is used as a sweetener. It has an antimicrobial effect on mutans streptococci [90]. The therapeutic effect of xylitol on caries arrest in children was seldom reported in the literature. A clinical study investigating the caries-arrest effect of xylitol was conducted in 510 children aged six years [91]. It was concluded that high-xylitol chewing gum was effective in arresting dentine caries if dental restoration could not be an option. However, a choking hazard in young children is a concern. Thus, the daily use of xylitol chewing gum is suggested as an effective adjunct for caries prevention for children older than five years old only [92]. Therefore, limited evidence exists to support the use of xylitol chewing gum or lozenges among preschool children [93]. 

Chlorhexidine (CHX) is an antibacterial agent that is commonly used as an antiseptic. It consists of cationic polybiguanide. At the physiologic pH, chlorhexidine salts are dissociated to yield positively charged chlorhexidine cations. The bactericidal effect is a result of the binding of this cationic molecule to negatively charged bacterial cell walls [94]. CHX has been studied for its potential in preventing and arresting caries [94,95]. Different concentrations and formulae (varnish, gel, toothpaste and mouthrinse) are available. A Cochrane review recently concluded that limited evidence exists to support or refute the better effectiveness of chlorhexidine when compared with the control group in decreasing the level of mutans streptococci or preventing dental caries in children [88]. Few studies demonstrated the effectiveness of chlorhexidine varnish in halting the progression of dental caries. A clinical study in children found that the combination of chlorhexidine varnish and fluoride varnish was more effective in enhancing the remineralisation of white spot lesions after three months than the separate application of the same agents [95]. So far, there have been no published studies using chlorhexidine in arresting dentine caries in preschool children.

CPP-ACP is one of the calcium-phosphate-based remineralization systems. It can provide calcium and phosphate ions as a reservoir to buffer plaque acidity (pH) and maintain the state of the supersaturation of tooth enamel, eventually enhancing the remineralisation process [96]. CPP-ACP can be incorporated into different products, such as chewing gums, mouthwashes and dental creams [97]. Recently, a systematic review concluded that CPP-ACP has a remineralising effect on early lesions in comparison with the control or placebo, even though this seems insignificantly different compared with that of fluorides [98]. The advantage of CPP-ACP is still ambiguous when it is used as a supplement to fluoridated dental products [98]. Up to now, no clinical trial has used CPP-ACP in arresting the progression of caries at the dentine level in preschool children. High-quality clinical trials are required to confirm the effectiveness of non-fluoride agents in controlling dental caries in preschool children.

## 4. Conclusions

Caries prevalence and severity in primary teeth of children are high. Untreated tooth decay in the primary dentition is a common and global phenomenon. The costly restorative treatment and the insufficient supply or skewed distribution of dental health workforce make it impracticable to control dental caries in children, especially in underprivileged communities. A simple program for promoting oral self-care via tooth brushing with fluoride dentifrice has been well documented with promising results. However, this program alone is probably not able to deal with severe cases involving multiple carious lesions. Evidence exists that fluoride varnish is considered a safe and effective agent for caries prevention in young children. For treating established dentine lesions, clinical trials of ART treatment with highly viscous GIC provide some evidence with good success rates in primary dentition. In light of its clinical success, SDF can be an option for controlling tooth decay, especially at the cavitation level in preschool children. SDF may revolutionize paediatric and community dentistry and may be a breakthrough dental agent this century due to its safety, efficiency, feasibility and effectiveness in preventing and arresting dentine caries. Although black staining is a known side effect of SDF, the health benefits of having no toothache and dental infection can far outweigh this, particularly where access to dental care is challenging. Regardless of various atraumatic approaches, good plaque control remains paramount for the success of caries control in young children.

## Figures and Tables

**Table 1:** Summary of systematic reviews on the effectiveness of atraumatic restorative treatment (ART) and silver diamine fluoride (SDF) in treating dental caries in children.

Authors, Year [Reference]	No. of Publications [No. of Patients]	Summary Findings
Van’t Hof et al., 2006 [32]	28 [NA]	Three-year survival rates for single-surface ART restorations in primary teeth using high-viscosity glass ionomer were 86%. Annual failure rates of multiple-surface restorations (17%) were higher than those of single-surface restorations (5%).
Mickenautsch et al., 2010 [33]	7 [NA]	No significant difference in the 2-year success rates of class I ART and amalgam restorations in primary teeth with the relative risks (RR 1.07; 95% CI: 0.91–1.27).
de Amorim et al., 2012 [30]	29 [NA]	The 2-year survival rates of single- and multiple-surface ART restorations in primary teeth were 93% (95% CI: 91–94%) and 62% (95% CI: 51–73%) respectively.
Duangthip et al., 2016 [20]	7 [594]	The use of minimally invasive approaches such as ART and hand excavation to preserve more tooth structure is beneficial for preschool children.
Tedesco et al., 2017 [31]	4 [NA]	No significant difference in survival rate between ART and conventional occluso-proximal restoration in primary molars (OR = 0.89, 95% CI: 0.57–1.37).
Rosenblatt et al., 2009 [42]	2 [827]	SDF’s lowest prevented fractions for caries arrest and caries prevention were 96% and 70% respectively. Sodium fluoride varnish’s highest prevented fractions for caries arrest and caries prevention were 21% and 56% respectively.
Peng et al., 2012 [43]	15 [NA]	Silver compounds are viable agents for preventing and arresting caries.
Duangthip et al., 2015 [44]	4 [967]	SDF applications or daily tooth brushing with fluoride toothpaste is effective in arresting caries in primary teeth of preschool children.
Gao et al., 2016 [45]	17 [NA]	5% sodium fluoride varnish can remineralise early enamel caries, and 38% SDF can arresting dentine caries. The overall proportion of arrested dentin caries by 38% SDF was 66% (95% CI: 41–91%).
Gao et al., 2016 [46]	19 [NA]	The overall percentage of caries arrest of 38% SDF in primary teeth was 81% (95% CI: 68–89%).
Contreras et al., 2017 [47]	7 [3073]	30% or 38% SDF shows potential as an alternative treatment for caries arrest in primary teeth.

NA = Number of included patients in a systematic review were not available. RR = relative risk. CI = confidence interval. OR = odds ratio.

**Table 2:** Summary of randomized clinical trials of atraumatic restorative treatment (ART) and silver diamine fluoride (SDF) conducted in preschool children.

Topic	Author, Year [Reference]	Duration, Place, and Participants	Intervention Group	Outcome
ART	Menezes et al., 2006 [48]	12 months, Brazil, 110 children, 4–6 years	(1) Vidrion: one-surface	Success rates of class I restorations between the two GIC materials were similar (Ketac Molar: 82% vs. Vidrion: 63%, *p* > 0.05). Success rates of class II restorations between the two GIC materials were not significantly different (Ketac Molar: 31% vs. Vidrion 18%, *p* > 0.05).
			(2) Ketac Molar: one-surface	
			(3) Vidrion: two-surface	
			(4) Ketac Molar: two-surface	
			(5) Vidrion: three- or four- surface	
			(6) Ketac Molar: three- or four-surface	
	Arrow et al., 2015 [49], Arrow, 2016 [29]	6–23 months, Australia, 254 children, mean 3.8 years	(1) ART technique	No significant difference in restorative success between groups after 12 months. More children in the control (49%) were referred for specialist care compared with ART (6%) (*p* < 0.001).
			(2) Conventional technique with local anaesthetic, rotary instruments and restoration (control)	
ART vs. SDF	Zhi et al., 2012 [50]	24 months, China, 212 children, 3–4 years	(1) 38% SDF once/yr (year)	Caries arrest rates of three groups were 79%, 91% and 82% respectively (*p* = 0.007).
			(2) 38% SDF twice/yr	
			(3) Flowable GIC filling once/yr	
	dos Santos et al., 2012 [51]	12 months, Brazil, 91 children, 5–6 years	(1) Interim GIC filling	Caries arrest rates of SDF and GIC filling were 67% and 39%, respectively (*p* < 0.001).
			(2) 30% SDF	
SDF	Chu et al., 2002 [52]	30 months, China, 375 children, 3–5 years	(1) Excavation + 38% SDF once/yr	The respective mean numbers of arrested caries tooth surfaces in the five groups were 2.5, 2.8, 1.5, 1.5 and 1.3, respectively (*p* < 0.001).
			(2) 38% SDF once/yr	
			(3) Excavation + 5% NaF 4 times/yr	
			(4) 5%NaF 4 times/yr	
			(5) Control	
	Fung et al., 2016 [53]	18 months, Hong Kong, 888 children, 3–4 years	(1) 12% SDF once/yr	Caries arrest rates in Groups 1 to 4 were 50%, 55%, 64%, 74%, respectively (*p* < 0.001).
			(2) 12% SDF twice/yr	
			(3) 38% SDF once/yr	
			(4) 38% SDF twice/yr	
	Duangthip et al., 2016 [54]	18 months, Hong Kong, 371 children, 3–4 years	(1) 30% SDF once/yr	Caries arrest rates of Groups 1 to 3 were 40%, 35% and 27%, respectively (*p* < 0.001).
			(2) 30% SDF three times weekly at baseline	
			(3) 5% NaF three times weekly at baseline	

Class I = restoration located on pit and fissure, on occlusal, buccal, and lingual surfaces of molars; Class II = restoration located on proximal surfaces of molars. GIC = glass ionomer cement.
